# Digitalisation, human factors, and mental health among primary care professionals aged ≥ 50 years: a mixed-methods case study in Portugal

**DOI:** 10.1186/s12913-026-14671-3

**Published:** 2026-05-22

**Authors:** Maria Piedade Brandão, Beatriz Chaves

**Affiliations:** 1 School of Health Sciences (ESSUA), University of Aveiro, Aveiro, Portugal; 2 RISE-Health, School of Health Sciences, University of Aveiro, Aveiro, Portugal; 3 Department of Social, Political and Territorial Sciences (DCSPT), University of Aveiro, Aveiro, Portugal

**Keywords:** Digitalisation, Technostress, Ageing workforce, Primary care, Mental health, Health professionals, Occupational wellbeing, Portugal

## Abstract

**Background:**

Digitalisation is transforming healthcare delivery but has introduced new psychosocial and organisational demands. Older workers may be particularly vulnerable to cognitive load, technostress, and reduced well-being during rapid digital transitions. However, evidence focused on primary care professionals aged ≥ 50 years remains limited. This study examined how workplace digitalisation is related to work organisation and mental health in this group.

**Methods:**

A mixed-methods case study was conducted in a personalised primary care unit in northern Portugal. A self-administered questionnaire was completed by 24 professionals aged ≥ 50 years (92% of eligible staff). Institutional documents were analysed to contextualise organisational policies. The quantitative data were analysed descriptively via group comparisons (χ² tests or exact tests, as appropriate), whereas the qualitative data from the open-ended responses and documents were examined via thematic content analysis. The findings were integrated through triangulation.

**Results:**

The participants recognised the benefits of digitalisation for organisational performance and efficiency (91.7%). However, 62.5% reported increased work-related stress, and 33.4% perceived a worsening of work–life balance. Only 25.0% felt that they had sufficient time to learn and adapt to new technologies. Among the respondents to the privacy-related items (*n* = 23), 60.9% agreed that the institution had clear data protection policies and that 60.9% felt confident about data security; however, only 26.1% reported receiving adequate training on data privacy (52.2% disagreed). The only significant difference by professional role concerned perceived time for adaptation: clinical staff more frequently disagreed that they had sufficient time (11/16, 68.8%), whereas nonclinical staff were mostly neutral (5/7, 71.4%) (Fisher–Freeman–Halton exact test, *p* = 0.003). The qualitative analysis identified four themes: digital acceleration and loss of autonomy; emotional fatigue and reduced human connection; learning inequalities by age and role; and collective coping and resilience. Documentary analysis revealed a stronger institutional emphasis on technological modernisation than on staff mental health.

**Conclusions:**

Digitalisation in primary care was experienced by older professionals as both an opportunity and a source of psychosocial strain. Without adequate training, protected learning time, participatory implementation, and explicit occupational mental health strategies, digital transformation may exacerbate stress and inequalities within an ageing workforce. Integrating human-centred design and age-sensitive training into digital health policies may support more sustainable and equitable primary care systems.

**Supplementary Information:**

The online version contains supplementary material available at 10.1186/s12913-026-14671-3.

## Background

Mental health is a core component of overall well-being and plays a central role in how individuals cope with daily demands, relate to others, and sustain performance at work [1]. In contemporary health systems, this dimension is increasingly challenged by rapid organisational and technological change [2]. The accelerating digitalisation of healthcare, encompassing electronic health records, online scheduling, telemedicine, and interoperable information systems, has reconfigured clinical practice, workflows, and professional relationships [3]. While these developments may enhance efficiency, data accuracy, and patient safety, they also introduce new human factor challenges, particularly when implementation is rapid, underresourced, or inadequately supported [4].

In this study, “mental health” is conceptualised as self-reported perceptions of work-related psychological strain, including stress, fatigue, and difficulties in maintaining work–life balance, rather than clinically assessed mental health conditions. This distinction is important given that the study focuses on subjective experiences of digital work environments rather than diagnostic outcomes. In parallel, the concept of “human factors” is understood as the interaction between individuals, technologies, tasks, and organisational contexts that shape performance, safety, and wellbeing in healthcare systems [4].

For healthcare workers, digital transformation can substantially alter task structures, cognitive load, and work–life boundaries. Poor system usability, fragmented digital workflows, and limited control over technological processes have been associated with technostress, frustration, and reduced job satisfaction [5, 6]. Technostress refers to stress arising from the use of information and communication technologies, particularly when technological demands exceed users’ coping capacities or available resources [4, 7]. When digitalisation is not accompanied by user-centred design, adequate training, and supportive leadership, it may contribute to heightened stress, perceived dehumanisation of care, and loss of professional autonomy [8, 9]. Within this perspective, psychosocial strain is seen as emerging from sociotechnical misalignment rather than from technology itself. These psychosocial risks are particularly salient in primary care and personalised care settings, where relational continuity, communication, and direct patient contact are central to professional identity and work organisation.

Older professionals represent a key population in this context. Although definitions vary, international organisations commonly identify workers aged 50 years and above as a group facing heightened vulnerability in the labour market [10, 11]. In healthcare, these professionals often combine extensive clinical experience with exposure to age-related stereotypes, accelerated technological change, and, in some cases, lower digital confidence or fewer training opportunities [12]. Rather than assuming age-related vulnerability per se, it is important to consider how organisational conditions—such as workload, access to training, and participation in decision-making—shape the experience of digitalisation among older workers. In the absence of targeted institutional support, workplace digitalisation may amplify experiences of overload, exclusion, and isolation, with potential consequences for mental health, work engagement, and workforce retention [8, 13].

Importantly, digitalisation does not inevitably harm wellbeing. When digital systems are developed and implemented according to human factor principles—emphasising usability, ergonomics, workflow integration, and participatory approaches—they may enhance perceived control, teamwork, communication, and psychological wellbeing [9, 14]. This perspective highlights that adverse outcomes are not inherent to technology itself but rather emerge from the interaction between humans, tasks, organisational contexts, and digital systems [4, 8, 9].

The COVID-19 pandemic further revealed the significance of these interactions. It accelerated the adoption of digital health solutions under crisis conditions, intensifying both their benefits and psychosocial risks [8, 13]. Within Portugal’s National Health Service, digital tools became indispensable for maintaining access to care and organisational coordination [15, 16]. For older professionals, the combination of increased workload and rapid digital acceleration may have exacerbated technostress and cognitive strain while also revealing systemic gaps in training, support, and occupational mental health strategies [6, 13].

Despite growing interest in digital health, empirical research focusing specifically on the human factors and mental health implications of workplace digitalisation among healthcare professionals aged 50 years and over remains limited, particularly with respect to older healthcare professionals in primary care and Southern European contexts [2, 8]. Existing studies often focus on digital skills or burnout in isolation without adequately examining how digital technologies intersect with work organisation, professional relationships, and psychosocial wellbeing in everyday clinical practice [2, 3, 8].

This study addresses this gap by examining how workplace digitalisation influences mental health, psychosocial wellbeing, and everyday clinical work among healthcare professionals aged 50 years and over in a Portuguese personalised primary care unit. Rather than assessing clinical mental health outcomes, the study focuses on perceived psychosocial impacts, including stress, workload, work–life balance, and organisational support. Using an integrated mixed-methods approach, the study explores professionals’ perceptions of digital tools and their implementation, self-reported psychosocial impacts, effects on work organisation and professional relationships, and perceived strategies to support digital adaptation and wellbeing within an ageing primary care workforce.

## Methods

### Design

This study employed a cross-sectional, exploratory mixed-methods case study design, combining quantitative and qualitative data to explore how digitalisation is experienced in relation to perceived psychosocial impacts in the workplace.

Mixed-methods approaches are widely recognised as effective for investigating complex psychosocial and organisational phenomena, allowing the integration of measurable patterns and subjective experiences [17].

In the present study, the qualitative component was designed to complement and contextualise the quantitative findings rather than to constitute a standalone in-depth qualitative inquiry. The quantitative data were collected through a self-administered online survey, and the qualitative data were derived from open-ended survey responses and documentary sources. Descriptive and inferential statistics were applied to the quantitative data, whereas thematic content analysis was used for the qualitative data.

### Setting

The study was conducted at a personalised primary care unit (*Unidade de Cuidados de Saúde Personalizados*, UCSP) in northern Portugal. Integrated within the National Health Service, the UCSP provides general practice, nursing, vaccination, and community health programmes to approximately 11,000 registered users. At the time of data collection, the unit employed 38 professionals—physicians, nurses, social workers, administrative staff, and operational staff—working under high workload and infrastructural constraints typical of the region’s healthcare context.

### Participants and eligibility criteria

The target population included all professionals employed at the UCSP-Chaves II.

The inclusion criteria were as follows:

1. Employment at the unit for ≥ 1 year;
2. Age 50 years or older;
3. Active engagement with digital systems in daily work;
4. Active employment during data collection;
5. Provision of informed consent.

Professionals on leave, retired, or primarily working outside the unit were excluded. Of the 26 professionals meeting these criteria, 24 agreed to participate (response rate: 92.3% of eligible staff; 63.2% of the total workforce). The sample included both clinical staff (physicians and nurses) and nonclinical staff (social workers, administrative staff, and operational staff), reflecting the unit’s multidisciplinary composition.

### Data collection

Data were collected between 1 May and 1 June 2025 via an online questionnaire hosted on the University of Aveiro’s institutional survey platform. The participants received an individual invitation email describing the study objectives, voluntary participation, and data protection measures, along with a secure survey link. The estimated completion time was approximately five minutes.

### Instruments

An ad hoc questionnaire comprising 28 items was developed for this study. It included:

- four sociodemographic items (professional role, gender, age, and length of service);
- twenty closed-ended items with five-point Likert scales (from strongly disagree to strongly agree) to assess perceptions of workplace digitalisation, work dynamics, institutional support, data privacy, and self-reported psychosocial impacts (e.g., stress, fatigue, and work–life balance);
- Four open-ended questions invited participants to describe how digitalisation affects their work, well-being, and professional relationships and to propose suggestions for improvement.

Instrument development was informed by the literature on occupational wellbeing, technostress, and digital transformation in healthcare [6, 8]. The questionnaire was reviewed by two domain experts—one with a PhD in epidemiology and a master’s degree in public health and one clinical psychologist—to ensure clarity, relevance to the target population, and adequate coverage of the study topics. Given the exploratory and context-specific nature of the study, no formal psychometric validation was conducted, as the instrument was intended to capture perceptions and experiences rather than to measure standardised psychological constructs [17, 18].

In addition, a documentary corpus was compiled to contextualise the organisational and policy environment of the unit. The documents analysed included (i) the Internal Regulation of the Local Health Unit of Trás-os-Montes e Alto Douro, which outlines the institution’s mission, values, and organisational structure, including the establishment of a Clinical Informatics Committee (2024); (ii) the 2023 Annual Report and Accounts of the same Local Health Unit, which describes institutional investment in technological modernisation, interoperability of information systems, equipment acquisition, and administrative process automation; and (iii) the 2020 Activity Report of the Northern Regional Health Administration, which contextualised the regional response to the COVID-19 pandemic and documented psychological support measures for both the general population and healthcare professionals. These documents were analysed via the same thematic content analysis principles applied to the qualitative survey data.

### Measures

Independent variables included sociodemographic and professional characteristics: professional function (grouped as clinical staff [physicians and nurses] and nonclinical staff [social workers, administrative personnel, and operational personnel]), sex, age group (50–60 years; >60 years), and length of service (≤ 20 years; >20 years).

The dependent variables were derived from closed-ended questionnaire items via five-point Likert scales and captured professionals’ perceptions across several domains. These included perceived digitalisation and use of digital tools in daily work; self-reported psychosocial impacts, such as stress, anxiety, fatigue, and low mood; perceived changes in work organisation and professional relationships; perceptions of innovation, leadership, and institutional support; and perceived adequacy of privacy and data protection practices.

All the dependent measures reflected subjective perceptions and experiences rather than clinical or psychological diagnoses. The selected domains are consistent with widely examined dimensions in research on technostress and organisational wellbeing, including technology-related stressors, work organisation, leadership, and institutional support [4, 6].

### Quantitative analysis

The quantitative data were analysed via IBM SPSS Statistics (version 30; IBM Corp., Armonk, NY, USA). Descriptive statistics, including absolute and relative frequencies, were used to characterise the sample and summarise the survey responses. For analytical purposes, five-point Likert-scale responses were collapsed into three categories: agree (combining strongly agree and agree), neutral (neither agree nor disagree), and disagree (combining strongly disagree and disagree). This approach was adopted to ensure adequate cell counts for categorical analyses.

Inferential analyses employed Pearson’s chi-square (χ²) test of independence to examine associations between categorical variables (e.g., professional group, age group, and length of service) and perceptions related to digitalisation and mental health. Yates’ continuity correction was applied in 2 × 3 contingency tables, and the Fisher–Freeman–Halton exact test was used when the expected cell counts were less than five [19]. Statistical significance was set at *p* < 0.05 (two-tailed).

The complete distribution of all the questionnaire items is provided in Supplementary Table S1.

### Qualitative analysis

Qualitative data from open-ended survey responses were analysed via thematic content analysis, following Bardin’s framework [20], which is widely applied in occupational and health research [21].

Although the qualitative data were limited in depth compared with full qualitative designs (e.g., interviews), they were used to provide contextual insights into participants’ experiences and to support interpretation of the quantitative findings.

The analytical process comprises three stages:

1. Preanalysis, involving selection and organisation of the textual corpus;
2. Material exploration, including the coding and grouping of meaning units into thematic categories;
3. Treatment and interpretation focus on the identification of patterns, silences, and contradictions relevant to the study aims.

The sentence was adopted as the unit of analysis to preserve contextual meaning. Categories were constructed on the basis of three criteria—semantic similarity, frequency of occurrence, and practical relevance—with particular attention to themes with implications for workplace improvement.

The same analytical principles were applied to the documentary corpus to examine how issues related to digitalisation and occupational mental health were addressed—or omitted—within institutional and policy documents.

### Integration of quantitative and qualitative data

A triangulation strategy was used to enhance validity and interpretative depth by integrating evidence from three data sources: (i) quantitative survey results, (ii) qualitative open-ended responses, and (iii) documentary analysis. Triangulation in this study involved comparing and contrasting findings across these data sources to identify convergences, complementarities, and inconsistencies, rather than achieving full methodological integration.

This approach allowed the qualitative and documentary data to contextualise and enrich the interpretation of survey findings within the organisational setting.

### Ethical considerations

The study complied with international and national ethical standards for research involving human participants. The protocol was reviewed and approved by the Ethics Committee of the *Centro Hospitalar de Trás-os-Montes e Alto Douro* (reference 5642). Authorisations were also obtained from the Board of Directors, the Ethics Committee, and the service director.

Participation was voluntary and anonymous. Before accessing the survey, participants viewed an information sheet describing the study aims, procedures, risks, benefits, and data protection in accordance with the European General Data Protection Regulation (GDPR) [22]. Only participants who provided electronic informed consent could proceed. No personally identifiable data were collected, and all procedures adhered to national data protection laws and institutional research ethics guidelines.

## Results

### Sample characteristics

Among the 26 eligible healthcare professionals aged ≥ 50 years, 24 participated in the study (92.3%). The sample included 16 clinical professionals (physicians and nurses), seven nonclinical staff members, and one participant who did not report their professional role. Most participants were women (91.7%).

Participants were aged 50–60 years (62.5%) or over 60 years (37.5%). Two-thirds of the sample (66.7%) reported a length of service exceeding 20 years, and most had worked at the same institution for more than 10 years (Table 1).

**Table 1:** Sociodemographic characteristics of participants (*N* = 24)

Characteristic	*n*	%
**Professional role**		
Clinical (physicians and nurses)	16	66.7
Nonclinical (social worker, administrative and operational staff)	7	29.2
Missing	1	4.2
**Gender**		
Female	22	91.7
Male	2	8.3
**Age group**		
50–60 years	15	62.5
> 60 years	9	37.5
**Length of service**		
1–5 years	1	4.2
6–10 years	2	8.3
11–20 years	5	20.8
> 20 years	16	66.7

Note. Percentages are based on the total sample (*N* = 24). One participant did not report their professional role

### Perceptions of digitalisation and work adaptation

Overall, the participants perceived digitalisation as necessary and beneficial for healthcare organisations (Table 2). Almost all the respondents agreed that digitalisation is important for organisational strategy (95.9%) and that digital tools improved the efficiency of work processes (91.7%).

However, perceptions of work adaptation were less favourable. Two-thirds of the participants (66.6%) agreed that digitalisation had increased their daily workload, whereas only one-quarter (25.0%) felt that they had sufficient time to learn and adapt to new technologies, with half of the respondents disagreeing.

These patterns were also described in qualitative responses, which provided additional context for the reported experiences.

**Table 2:** Perceptions of digitalisation, work adaptation and wellbeing (*N* = 24)

Domain	Item	Agree (%)	Neutral (%)	Disagree (%)
Perceived benefits of digitalisation	Digitalisation is important for healthcare organisations	95.9	0.0	4.2
	Digital tools improved efficiency of work processes	91.7	4.2	4.0
Work adaptation and workload	Digitalisation increased my daily workload	66.6	20.8	12.5
	I have enough time to learn and adapt to new technologies at work	25.0	25.0	50.0
Mental health and wellbeing	The introduction of new technologies increased my work-related stress	62.5	20.8	16.7
	Digitalisation negatively affects work–life balance	33.4	29.2	37.5
Leadership and participation	Leadership supports the digital transition	50.0	33.3	16.6
	I was consulted in the choice of digital tools	20.8	16.7	62.5
Organisational culture and support	Mental health is a priority in organisational culture	37.5	16.7	45.8
Data protection and privacy	I feel confident that patient data are secure in the digital systems I use	60.8	17.4	21.7
	I received adequate training on patient data privacy	26.1	21.7	52.1

Note. Percentages may not total 100 due to rounding. Five-point Likert responses were collapsed into three categories: agree (combining strongly agree and agree), neutral (neither agree nor disagree), and disagree (combining strongly disagree and disagree)

### Impact on mental health and wellbeing

Digitalisation was frequently reported as being associated with self-reported psychosocial strain (Table 2). Nearly two-thirds of the participants (62.5%) agreed that the introduction of new technologies had increased work-related stress, and one-third (33.4%) reported a negative impact on their work–life balance.

Open-ended responses further illustrated these experiences, with participants referring to “technological overload,” “fear of making mistakes,” and a perceived “loss of control,” providing additional context for the quantitative findings.

### Work organisation, leadership, and team dynamics

Perceptions of leadership and participation in digital change were mixed (Table 2). While half of the participants (50.0%) agreed that leadership supported the digital transition, only 20.8% reported having been consulted in the choice of digital tools, with nearly two-thirds (62.5%) disagreeing.

Qualitative findings reflected these patterns, with participants describing both positive views of digitalisation as a marker of innovation and concerns related to increased workload, reduced autonomy, and limited involvement in decision-making.

### Institutional culture and perceived support

Institutional attention to staff mental health was perceived as limited (Table 2). Fewer than four of the ten participants (37.5%) agreed that mental health was considered a priority within organisational culture, whereas almost half disagreed.

Participants highlighted the need for structured training, protected learning time, and more proactive technical and psychological support. Training was commonly described as sporadic and occurring after new digital systems had already been implemented.

### Data protection and privacy awareness

Confidence in data protection practices was moderate (Table 2). While 60.8% of the participants felt confident that patient data were secure in the digital systems they used, only 26.1% reported having received adequate training on data privacy, and more than half disagreed that such training had been sufficient.

Qualitative responses indicated uncertainty in applying data protection practices, particularly when using newly introduced or remote digital systems.

### Group differences

Perceived time to learn and adapt to new technologies differed significantly by professional role (Fisher–Freeman–Halton exact test, *p* = 0.003). Clinical staff more frequently disagreed that they had sufficient time (11/16, 68.8%), whereas most nonclinical staff reported a neutral position (5/7, 71.4%). No other comparisons by professional role reached statistical significance.

### Qualitative findings

Findings from qualitative responses and documentary analysis were examined alongside quantitative results to identify convergent and divergent patterns.

### Perceived impacts and organisational responses

Thematic analysis of the open-ended responses revealed several interrelated themes reflecting both the perceived impacts of digitalisation and the participants’ expectations for organisational responses (Table 3).

Across all the questions, digital skills and training emerged as central concerns. Participants reported insufficient initial and ongoing training, frequent reliance on self-learning, and perceived learning inequalities.

**Table 3:** Perceived impacts of digitalisation and suggested organisational responses

Theme	Key issues identified by participants	Illustrative quotes
Digital skills and training	Lack of initial and ongoing training; reliance on self-learning; learning inequalities by age	“The lack of training is at the root of all difficulties.”
		“We often face new systems without knowing how to use them.”
Technological infrastructure	Outdated equipment; slow or unstable systems; poor technical support	“The equipment should be improved.”
		“Systems are slow and delay our work.”
Usability and workload	Complex navigation; excessive number of steps; duplication of records	“The system is not intuitive.”
		“The number of steps makes everything slower.”
Work organisation and time pressure	Lack of time for clinical care; overload of administrative tasks	“Digitalisation takes time away from patients.”
		“There is not enough time for everything.”
Psychosocial strain	Stress, fatigue, frustration, anxiety about errors	“It causes exhaustion.”
		“When systems fail, my stress increases.”
Relational care and communication	Reduced patient interaction; less team communication	“Digitalisation distances us from patients.”
Adaptation and resilience	Peer support; informal learning; professional commitment	“We help each other when new systems come in.”
Expectations for improvement	Need for training, better equipment, and participatory implementation	“We should be trained before new systems are introduced.”

Concerns regarding technological infrastructure were also prominent, including outdated equipment, slow or unstable systems, and limited technical support. These issues were associated with reported challenges in system usability and workload, including complex navigation, multiple documentation steps, and duplication of records.

Participants also described challenges related to work organisation and time pressure, including reduced time available for direct patient care and increased administrative demands. These experiences were accompanied by reports of stress, fatigue, and frustration.

Despite these challenges, participants described forms of adaptation, including peer support, informal learning, and professional commitment. Expectations for improvement focused on structured training, improved equipment, and greater participation in digital implementation processes.

### Documentary analysis

Analysis of institutional documents revealed a strong emphasis on technological modernisation, efficiency, and digital performance indicators. Strategic and regulatory texts focused predominantly on system implementation, interoperability, and productivity gains, with little attention given to the human and psychosocial dimensions of digital work.

No explicit provisions addressing staff mental health, psychosocial risk prevention, or the wellbeing implications of digitalisation were identified in routine organisational documents. References to psychological support appeared primarily in documents related to the COVID-19 pandemic, indicating that such issues were addressed in specific contexts rather than systematically across policies.

#### Work–life balance

Perceptions of the impact of digitalisation on work–life balance were mixed. Some participants reported that improved access to patient records and electronic documentation improved efficiency and reduced the time spent on certain administrative tasks.

However, negative experiences were more frequently reported. Participants described physical and emotional fatigue, extended working hours, and reduced time available for direct patient care. Several noted that the need to complete digital records outside scheduled working hours blurred the boundaries between professional and personal life.

Qualitative responses highlighted increased cognitive demands associated with continuous interaction with digital systems, which participants associated with a perceived imbalance between work demands and personal wellbeing.

## Discussion

This mixed-methods case study examined how digitalisation is perceived to affect the psychosocial wellbeing and daily work experiences of healthcare professionals aged 50 years and over in a Portuguese personalised primary care unit. Overall, the findings depict a paradoxical scenario: digitalisation is widely recognised as necessary and beneficial for efficiency, information access, and care modernisation, yet it is also experienced as a significant source of stress, emotional fatigue, and perceived loss of control—particularly among frontline clinical staff. These findings should be interpreted as context-specific perceptions within a single organisational setting rather than as generalisable evidence. This ambivalence is consistent with previous research examining the psychosocial implications of digital transformation in healthcare settings [5, 8, 9].

### Digitalisation, work adaptation, and the ageing workforce

The sociodemographic profile of the sample reflects broader European trends, characterised by a predominantly female and ageing healthcare workforce [23]. Most participants reported long tenure within the same institution, with two-thirds of the sample having more than 20 years of service. This indicates that a stable workforce is now facing accelerated technological change, often within organisational contexts marked by limited resources. Such configurations may intensify tensions between accumulated professional experience and the need for rapid adaptation to evolving digital systems, particularly in primary care environments [8, 11, 24].

Differences by professional role were observed most clearly in relation to perceived time to learn and adapt to new technologies. Only one quarter of the participants reported having sufficient time for digital adaptation, and clinical staff were significantly more likely than nonclinical staff were to report time constraints. In addition, qualitative findings suggest higher levels of fatigue, anxiety, and perceived digital burden among clinicians, reinforcing evidence that digital transformation does not affect all professional groups equally [8, 24]. Importantly, given the absence of a comparison group, these findings do not allow conclusions about age-specific vulnerability but rather highlight how digitalisation is experienced within an older workforce in this organisational context. Together, these findings underscore the importance of organisational conditions—rather than age alone—in shaping digital adaptation among older professionals.

### Technostress and mental health in digitalised primary care

The results support existing evidence that digitalisation can contribute to technostress, understood as a constellation of stress, fatigue, and cognitive overload associated with technological demands [6, 7]. In this study, nearly two-thirds of the participants reported increased work-related stress linked to the introduction of new technologies, whereas qualitative responses frequently described experiences of “digital tiredness,” frustration with nonintuitive systems, and anxiety related to potential errors. These experiences echo mechanisms previously identified in healthcare settings, including information overload, time pressure, and reduced perceived autonomy [4, 5, 9].

Importantly, these difficulties were attributed not to age per se but to organisational factors, including high workload (reported by two-thirds of the participants), limited time for learning and adaptation, insufficient training, and a lack of participatory implementation. Similar patterns have been reported in primary care contexts where digital acceleration has not been matched by adequate organisational support, particularly for older workers [5, 8].

Taken together, these findings suggest that perceived psychosocial strain associated with digitalisation may be better understood as a function of sociotechnical misalignment rather than as an inherent consequence of technological change.

### Institutional culture, support, and organisational responsibility

Perceived gaps in institutional support were especially evident in relation to data protection practices. Although approximately 60% of the participants expressed confidence in the security of patient data, fewer than one-third reported having received adequate training on how to ensure data privacy in daily practice. This finding aligns with usability research showing that effective compliance with digital governance requirements depends heavily on practical, context-specific training rather than policy awareness alone [4].

Documentary analysis reinforced these perceptions. Institutional documents strongly emphasised efficiency, technological modernisation, and system interoperability while largely omitting explicit references to staff mental health, psychosocial risk prevention, or the wellbeing implications of digital work. References to psychological support appeared primarily in documents produced during the COVID-19 pandemic, suggesting that workforce mental health has been framed as a crisis-related concern rather than an integral component of a routine digital strategy.

This contrast between organisational discourse and frontline experiences highlights a potential disconnect between the strategic framing of digitalisation as a technical process and its lived psychosocial implications for healthcare workers. This misalignment indicates that digitalisation is predominantly considered a technical and efficiency-driven process, with limited integration of human and psychosocial considerations into organisational planning [8, 13].

### Work organisation, leadership, and relational care

The findings also reveal an ambivalent impact of digitalisation on the relational and communicative aspects of care. While a substantial proportion of participants perceived improvements in information flow and coordination, others reported that digital systems reduced opportunities for direct patient interaction and informal communication within teams. These contrasting experiences mirror previous studies examining the effects of electronic documentation and digital systems on relational care and professional interaction [5, 9].

This ambivalence extended to work–life balance. One-third of the participants reported that digitalisation negatively affected their work–life balance, and qualitative accounts highlighted blurred boundaries between professional and personal life, particularly when digital documentation was completed outside scheduled working hours. These experiences are consistent with research showing that constant digital connectivity can intensify emotional exhaustion and reduce opportunities for psychological recovery [2, 13].

Within this study, such effects were perceived rather than objectively measured and should therefore be interpreted as subjective experiences of digital work organisation. For older professionals whose professional identity is closely linked to relational and person-centred care, the perceived erosion of human connection may be especially distressing [10, 11].

### Practical implications for organisations and policy

The findings point to several practical implications at both the organisational and policy levels. At the organisational level, digitalisation initiatives should be accompanied by structured and ongoing training adapted to varying levels of digital literacy, with protected time allocated during working hours. This is particularly important given that only one quarter of the participants felt that they had sufficient time to learn and adapt to new technologies. Investments in updated infrastructure and equipment are also critical to reduce frustration, inefficiency, and error risk. In addition, accessible technical and psychological support services, alongside mechanisms for meaningful worker participation—such as feedback loops and codesign processes—may help mitigate technostress and enhance acceptance of digital change [8].

Crucially, institutional digital strategies should explicitly integrate mental health considerations, including psychosocial risk assessment and monitoring. In this study, fewer than four in ten participants agreed that mental health was a priority within organisational culture, indicating a substantial gap between digital innovation efforts and workforce wellbeing.

These implications should be interpreted as context-informed recommendations derived from participants’ experiences rather than as evidence-based interventions tested within this study. Within this context, digital transformation may contribute to or exacerbate existing inequalities and shift the burden of adaptation onto individual workers [25].

At the policy level, the findings support calls for more integrative frameworks linking digital innovation with workforce wellbeing. International organisations such as the WHO and OECD have highlighted the need for age-sensitive, inclusive digital strategies that promote both system performance and occupational health [11, 26]. While the present study does not provide generalisable evidence, it offers contextually grounded insights that may inform future policy and research in this area.

### Strengths and limitations

This study has several strengths, including the triangulation of quantitative, qualitative, and documentary data and its focus on an underresearched population: older healthcare professionals in primary care. The high response rate among eligible participants enhances the credibility of the findings within the case context.

However, limitations must be acknowledged. The single-case design and small sample size limit generalisability and statistical power. The cross-sectional nature of the study precludes causal inference, and the reliance on self-reported data may introduce recall or response bias.

In addition, the qualitative component, based on open-ended survey responses, provides limited depth compared with in-depth qualitative methods, and should therefore be interpreted as complementary rather than comprehensive.

In addition, the interchangeable use of the terms “digitalisation” and “digital transformation” in the questionnaire may have introduced conceptual ambiguity. Finally, the limited attention to mental health in institutional documents restricts the depth of policy analysis; however, this absence itself constitutes an important finding, highlighting a gap in organisational discourse rather than merely a limitation of the data [20].

### Future research

Future research should extend this work by examining multiple organisational contexts or conducting cross-regional comparisons on the basis of levels of digital maturity. Longitudinal designs would be particularly valuable for capturing how perceptions, coping strategies, and health outcomes evolve over time [27].

In addition, in-depth qualitative approaches (e.g., interviews or focus groups) could provide a more detailed understanding of the mechanisms underlying the perceptions identified in this study, including issues related to identity, professional roles, and adaptation to digital change.

Intervention studies are also needed to evaluate the effectiveness of age-sensitive training, participatory design approaches, and digital wellbeing programs in reducing technostress and improving occupational health [8].

## Conclusion

Digitalisation in primary care is experienced by older healthcare professionals as both an opportunity and a source of psychosocial strain. While most participants acknowledged gains in efficiency, accessibility, and coordination, a substantial proportion also reported stress and mental fatigue, with nearly two-thirds indicating increased work-related stress and many describing difficulties keeping pace with rapid technological change. These tensions appear to be shaped not by individual ageing alone but by organisational and systemic choices related to training, workload, participation in decision-making, and institutional support.

The findings should be interpreted as exploratory and context-specific, reflecting perceptions within a single primary care setting.

They also point to a gap between institutional agendas centred on technological modernisation and the limited integration of mental health considerations into digital strategies. Addressing this gap may require greater alignment between technological implementation and workforce support, although further research is needed to confirm these patterns across settings.

A more humane and inclusive digital transition requires the recognition of workforce mental health as a core dimension of digital governance. Aligning technological innovation with the lived experiences, capacities, and vulnerabilities of older professionals may contribute to more sustainable, high-quality primary care and to the promotion of wellbeing in an ageing health workforce.

## Electronic Supplementary Material

Below is the link to the electronic supplementary material.

Supplementary Material 1

## Data Availability

The datasets generated and/or analysed during the current study are not publicly available owing to the small sample size and the risk of indirect identification of participants but are available from the corresponding author upon reasonable request, subject to institutional approval and data protection requirements. The questionnaire and the qualitative coding framework are available from the corresponding author upon reasonable request.
