# Supplementary material for: Digitalisation, human factors, and mental health among primary care professionals aged ≥ 50 years: a mixed-methods case study in Portugal
Source: BMC Health Serv Res. 2026 May 22;26:1000. doi: 10.1186/s12913-026-14671-3 (PMC13386701; doi:10.1186/s12913-026-14671-3)
Supplement: Supplementary file 1 — Supplementary Material 1 [file 12913_2026_14671_MOESM1_ESM.docx]

| **Table S1. Full distribution of questionnaire items (collapsed Likert categories).** | | | | |
| --- | --- | --- | --- | --- |
| **Domain** | **Item** | **Agree (%)** | **Neutral (%)** | **Disagree (%)** |
| **Digital transformation** | Digital transformation influences the performance of healthcare organisations. | 91.7 | - | 8.4 |
|  | I consider digital transformation important in the strategy of healthcare organisations. | 95.9 | - | 4.2 |
|  | I adapt easily to new technologies introduced in my work environment. | 62.5 | 29.2 | 8.3 |
|  | The digital tools I use in my work are easy to use. | 70.8 | 8.3 | 20.8 |
|  | Digitalisation has improved the efficiency of processes in my work. | 91.7 | 4.2 | 4.0 |
| **Impact on mental health and well-being** | The introduction of new technologies has increased my stress levels at work. | 62.5 | 20.8 | 16.7 |
|  | I feel comfortable with the amount of information I need to manage due to digitalisation. | 54.2 | 20.8 | 25.0 |
|  | Digitalisation has made it more difficult to balance my personal and professional life. | 33.4 | 29.2 | 37.5 |
|  | I feel that there are generational differences in adapting to new technologies at work. | 87.5 | 8.3 | 4.2 |
|  | The introduction of new digital tools contributes to reducing my mental fatigue. | 33.3 | 29.2 | 37.5 |
| **Work dynamics and interactions** | Digital transformation fosters good relationships among healthcare teams. | 33.4 | 37.5 | 29.1 |
|  | Digital technologies have improved communication with patients. | 41.6 | 16.7 | 41.6 |
|  | Digitalisation has increased my daily workload. | 66.6 | 20.8 | 12.5 |
|  | I feel that I have enough time to learn and adapt to new technologies at wor | 25.0 | 25.0 | 50.0 |
|  | Digitalisation has reduced repetitive tasks and freed up time for other activities. | 50.0 | 25.0 | 25.3 |
| **Innovation and Leadership** | Digitalisation has encouraged innovation in my professional practice. | 58.4 | 20.8 | 20.8 |
|  | Leadership in my workplace supports the transition to digitalisation. | 50.0 | 33.3 | 16.6 |
|  | Leadership in my workplace is a critical factor in the implementation of digital transformation in healthcare. | 33.3 | 50.0 | 16.7 |
|  | I have participated in or been consulted on the selection of digital tools implemented in my workplace. | 20.8 | 16.7 | 62.5 |
| **Organisational culture and mental health support** | Mental health is considered a priority in the organisational culture of my institution. | 37.5 | 16.7 | 45.8 |
|  | I feel that the institution cares about the psychological well-being of its staff. | 20.9 | 20.8 | 58.3 |
|  | I am aware of and have access to my institution’s mental health policies. | 16.7 | 25.0 | 58.4 |
|  | The technical support available is sufficient to resolve issues related to digital tools. | 12.5 | 37.5 | 50.0 |
|  | I feel that I can share mental health concerns with my superiors. | 25.0 | 33.3 | 42.0 |
|  | My team openly discusses the challenges associated with digitalisation. | 12.5 | 37.5 | 50.0 |
| **Perception of Data Privacy** | I am confident that patient data is secure in the digital systems I use. | 60.8 | 17.4 | 21.7 |
|  | The institution has clear policies on protecting patient data privacy. | 60.8 | 21.7 | 17.4 |
|  | I have received adequate training on how to ensure the privacy of patient data. | 26.1 | 21.7 | 52.1 |

Note. Percentages may not total 100 due to rounding.
